# Design and development of a self-assembling protein nanoparticle displaying PfHAP2 antigenic determinants recognized by natural acquired antibodies

**DOI:** 10.1371/journal.pone.0274275

**Published:** 2022-09-12

**Authors:** Farhad Zahedi, Akram Abouie Mehrizi, Soroush Sardari, Iran Alemzadeh

**Affiliations:** 1 Malaria and Vector Research Group, Biotechnology Research Center, Pasteur Institute of Iran, Tehran, Iran; 2 Chemical and Petroleum Engineering Department (BBRC), Sharif University of Technology, Tehran, Iran; 3 Drug Design and Bioinformatics Unit, Medical Biotechnology Department, Biotechnology Research Center, Pasteur Institute of Iran, Tehran, Iran; Ehime Daigaku, JAPAN

## Abstract

**Backgrounds:**

In order to move towards the elimination and eradication of malaria in the world, the development of vaccines is inevitable. Many modern vaccines are based on recombinant technology; however, they may not provide a fully protective, long-lasting immune response. One of the strategies to improve recombinant vaccines is designing the nanovaccines such as self-assembling protein nanoparticles (SAPNs). Hence, the presentation of epitopes in a repeat array and correct conformation should be considered. *P*. *falciparum* generative cell-specific 1 (PfGCS1) is a main transmission-blocking vaccine candidate with two highly conserved fragments, HAP2-GCS1 and cd loop, inducing partial malaria transmission inhibitory antibodies. Therefore, to design an effective malaria vaccine, we used cd loop and HAP2-GCS1 fragments at the amino and carboxy terminuses of the SAPN-forming amino acid sequence, respectively.

**Methodology/Principal findings:**

The SAPN monomer (PfGCS1-SAPN) sequence was designed, and the three-dimensional (3D) structure was predicted. The result of this prediction ensured the presence of antigens on the SAPN surface. Then the accuracy of the predicted 3D structure and its stability were confirmed by 100 ns molecular dynamics (MD) simulation. The designed SAPN substructure sequence was synthesized, cloned, and expressed in *Escherichia coli*. With a gradual decrease in urea concentration in dialysis solutions, the purified proteins progressed to the final desired structure of the SAPN, which then was confirmed by Dynamic Light Scattering (DLS) and Field Emission Scanning Electron Microscopy (FESEM) tests. According to the Enzyme-Linked Immunosorbent Assay (ELISA), antigenic determinants were presented on the SAPN surface and interacted with antibodies in the serum of malaria patients.

**Conclusions/Significance:**

Our results show that the SAPN formed by PfGCS1-SAPN has produced the correct shape and size, and the antigenic determinants are presented on the surface of the SAPN, which indicates that the designed SAPN has great potential to be used in the future as a malaria vaccine.

## Introduction

Malaria, one of the most important infectious diseases in the world, is caused by six species of *Plasmodium*, belonging to the *Apicomplexa* phylum, and transmitted by the bites of Anopheles female mosquito species [1, 2]. The World Health Organization (WHO) reported an estimated 241 million malaria cases in 2020, with 627,000 deaths during the same period [3]. The development of radical and new tools, including genetically modified vectors, drugs, and vaccines, is required to eliminate and eradicate malaria. Due to the environmental problems of insecticides and resistance of mosquitoes to them, the resistance of *Plasmodium* parasites to various drugs, and the emergence of new behaviors in the vector, to maintain a level of recent disease control and move towards the elimination and eventual eradication of malaria, vaccine development is inevitable [4–6].

Although traditional vaccines are effective, they may cause infections, allergies, and autoimmunity [7, 8]. Modern vaccines have therefore shifted towards the use of subunit vaccines as recombinant vaccines [7]. Current recombinant vaccines do not provide a fully protective, long-lasting immune response; even the effectiveness of the RTS,S vaccine as a leading recombinant malaria vaccine is about 40% in children aged 5 to 17 months [9, 10]. Subunit vaccines, unlike pathogens, are not particulate and therefore have low immunogenicity. Hence, the presentation of epitopes in a repeat array and correct conformation can stimulate strong and protective immune responses [11]. In this regard, nanovaccines can be an appropriate platform [12]. Nanovaccines, microscopic particles with very high surface area to volume ratio and at least one dimension between 1 and 100 nm in size, are a new group of vaccines that have been developed by combining epitopes into nanoparticles to stimulate both humoral and cell-mediated immune responses. They have common characteristics with pathogens, such as size, shape, and pathogen-associated molecular patterns (PAMPs), that make them highly effective and improve their antigen stability, immunization, and performance [13, 14].

Self-Assembling Protein Nanoparticle (SAPN) is an approach used to improve the vaccine efficiency of subunit vaccines, especially for small particles (<10 nm), to assemble the particles into larger particles to make a suitable presenting system [15–17]. SAPNs are suitable carriers for vaccines due to their particulate repetitive antigen display characteristics [18, 19]. SAPN substructure consists of a pentameric and a trimeric coiled-coil oligomerization domain. There is a glycine-glycine linker between these two domains to join them with flexibility [20]. The oligomerization domains form multiple coiled-coil structures after refolding and cause the construction of spherical SAPNs [21]. The architecture of the SAPN substructure is such that the amino and carboxyl terminuses of the amino acid sequence locate on the SAPN surface, which subsequently exposes the antigens embedded in these terminals [22]. Kaba et al. designed a vaccine containing *Plasmodium berghei* circumsporozoite repeat epitope using the SAPN platform, which confers a long-lasting protective immune response to mice [22].

One of the antigenic candidates for the malaria vaccine is the *Plasmodium falciparum* generative cell-specific 1 (PfGCS1) antigen, which is on the plasma membrane of *P*. *falciparum* male gametes and gametocytes. Knocking down the *gcs1* gene blocks malaria transmission by preventing membrane fusion required for successful fertilization of the sexual stages of the parasite [23, 24]. HAP2-GCS1 and cd loop are two highly conserved fragments of this antigen that induce the production of malaria transmission inhibitory antibodies [25–29]. Concerning partial transmission blocking activity of the raised antibodies to PfGCS1 by recombinant vaccines [30, 31], a new presentation of these domains on a SAPN may help to increase the efficacy of the PfGCS1-based vaccine. Therefore, to design an effective malaria vaccine, we used the cd loop and HAP2-GCS1 domains of PfGCS1 antigen at the amino and carboxy terminuses of the SAPN-forming amino acid sequence, respectively, and to enhance the immunogenicity of the designed SAPN, the pan-allelic DR epitope (PADRE) was included in this structure. PADRE is a universal CD4+ epitope that targets immunologically diverse human populations and has the ability to bind to approximately 87% of known receptors encoded by the HLA-DR cluster of genes [32–34].

In this investigation, we designed a nanovaccine candidate molecule for malaria using the SAPN platform and evaluated it *in silico* and *in vitro*. After SAPN substructure sequence design, the predicted three-dimensional (3D) structure ensured the presence of antigens on the SAPN surface. Then the accuracy of the predicted 3D structure and its energy stability were confirmed by a 100 ns molecular dynamics (MD) simulation. After assuring the correct assembly of the SAPN substructures using bioinformatics, the gene was synthesized, cloned, and expressed in *Escherichia coli*. By gradual decrease in urea concentration in dialysis solutions, the purified peptides progress to the final structure of the SAPN, which then was confirmed by Dynamic Light Scattering *(*DLS), and Field Emission Scanning Electron Microscopy (FESEM) tests. According to the Enzyme-Linked Immunosorbent Assay (ELISA), antigenic determinants were presented on the SAPN surface and interacted with antibodies in the serum of malaria patients.

## Materials and methods

### Designing SAPN substructure (PfGCS1-SAPN)

**Fig 1:**
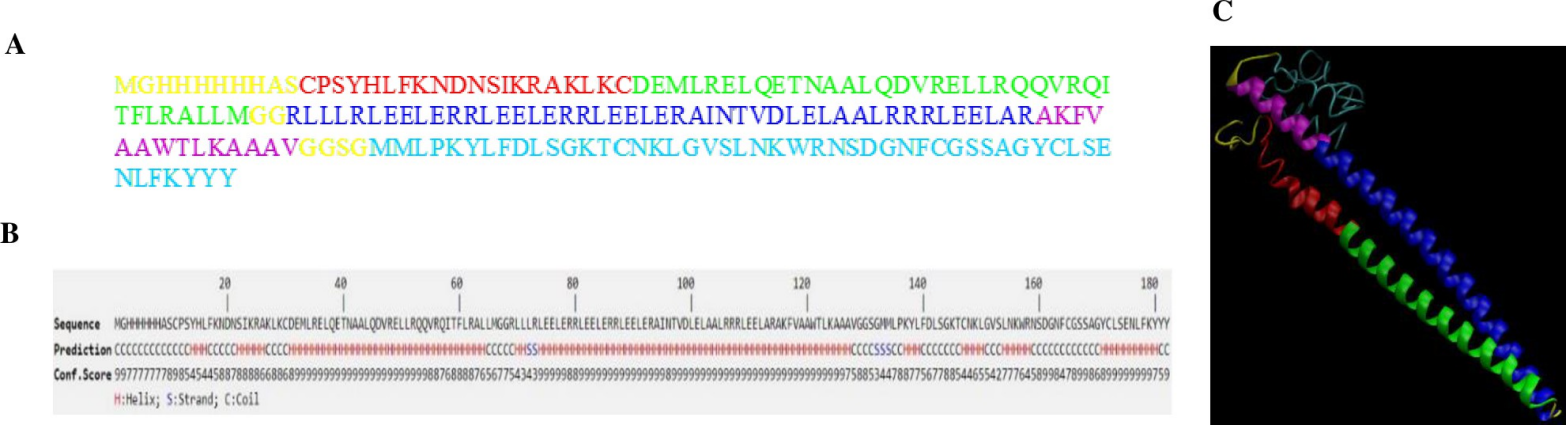
The amino acid sequence, secondary and tertiary model of PfGCS1-SAPN. (**A**) The amino acid sequence of the PfGCS1-SAPN. (**B**) Secondary structure of the designed PfGCS1-SAPN sequence. (**C**) Tertiary model of PfGCS1-SAPN after prediction by Quark server. This picture is represented by VMD 1.9.3. The color-coding used to make the picture is as follow: **Yellow**: His-tag and linkers, **Red**: cd loop, **Green**: pentamer, **Blue**: trimmer, **Magenta**: universal CD4 PADRE T-helper epitope, **Cyan**: HAP2-PfGCS1 domain.

The antigenic parts of the sequence were designed based on immunogenic domains of the PfGCS1 antigen and PADRE T-helper epitope for the intended SAPN substructure named PfGCS1-SAPN (accession number: OM937277). Two fragments of the PfGCS1 antigen called cd loop and HAP2-PfGCS1 domain were selected and placed on the N-terminus and the C-terminus sides of the PfGCS1-SAPN basis, respectively. The PfGCS1-SAPN base comprises pentameric and trimeric domains connected with two glycines as the linker. The pentameric and trimeric domains in order to have the ability to self-assemble, were selected based on the best pentameric and trimeric sequences in the literature [21, 35]. Self-assembling property was determined based on the coiled-coil structure formation of pentameric and trimeric domains and the way PfGCS1-SAPN 3D structure poses to interact with each other. The two features of the presence of coiled-coil components and the best desired 3D shape of the PfGCS1-SAPN basis among the sequences in the literature were predicted by the ExPASy COILS program (https://embnet.vital-it.ch/software/COILS_form.html) and Quark server (https://zhanglab.dcmb.med.umich.edu/QUARK), respectively. Six histidines were also used at the amino terminus to identify the peptide and facilitate the protein purification. The PfGCS1-SAPN amino acid sequence is shown in Fig 1A.

### Physicochemical parameters, antigenicity, and allergenicity evaluation of the PfGCS1-SAPN

The PfGCS1-SAPN physicochemical parameters were calculated by the ExPASy ProtParam tool (https://web.expasy.org/protparam), and the solubility characteristic was measured by the Protein-Sol server (https://protein-sol.manchester.ac.uk/results/solubility/run-16c4876302b939150250/results.html). Then, the antigenicity and allergenicity of PfGCS1-SAPN were detected using the VaxiJen (http://www.ddg-pharmfac.net/vaxijen/VaxiJen/VaxiJen.html) and the AlgPred servers (https://webs.iiitd.edu.in/raghava/algpred), respectively. These *in silico* antigenicity and allergenicity evaluation results serve as a hopeful prediction for future studies.

### Validation of the presence of coiled-coil fragments in PfGCS1-SAPN

The presence of coiled-coil forming parts of the designed amino acid sequence was evaluated by the ExPASy COILS program (https://embnet.vital-it.ch/software/COILS_form.html). For checking the number of homomers that each coiled-coil domain formed, they were uploaded to the GalaxyHomomer server (http://galaxy.seoklab.org/cgi-bin/submit.cgi?type=HOMOMER), separately.

### *In silico* prediction of PfGCS1-SAPN 3D model

The tertiary structure of PfGCS1-SAPN was predicted by the QUARK server (https://zhanglab.dcmb.med.umich.edu/QUARK). In CASP9 and CASP10 experiments, the QUARK server was ranked first in Free-modeling (FM). This server also provided the confidence score for each amino acid along with the secondary structure of the PfGCS1-SAPN. The protein secondary structure by the QUARK server was reported for the predicted 3D structure to specify what secondary structure the PfGCS1-SAPN acquired. The confidence score values ranged from 0 (least confident) to 9 (most confident) for predicting secondary structure.

### MD simulation

The MD simulation was performed for 100 ns on the Unix platform for data collection by GROMACS 2020. The charmm36 was used to act as the force field. The system energy was minimized for 50,000 steps of 1 fs by the steepest descent method to achieve the local minimum of the structure after placing the initially predicted peptide in the neutralized net charge box containing water and NaCl ions. Temperature and pressure equilibrated for a total time of 100 ps each to reach the target value of 300 K and 1 bar, respectively, to stabilize the solvent and ions around the peptide and relax the system.

### Validation of the predicted 3D structure of PfGCS1-SAPN and assembly of the stabilized simulated PfGCS1-SAPN

First, two analyses of root-mean-square deviation (RMSD) and radius of gyration (Rg) were performed by GROMACS 2020 concerning the initially predicted PfGCS1-SAPN 3D structure to ensure the accuracy and stability of the predicted 3D structure. Then, the structures obtained during the MD were clustered by the GROMOS method in GROMACS 2020 to find the cluster center of the most populated cluster as representative of the PfGCS1-SAPN after MD simulation. The Ramachandran plots of predicted and simulated PfGCS1-SAPN 3D structure were calculated by the MolProbity server (http://molprobity.biochem.duke.edu), separately to compare and validate the correction of the peptide 3D structure after MD simulation. The superimposition of the simulated 3D structure and predicted PfGCS1-SAPN 3D structure was performed by PyMOL v.2.3.0 to compare the simulated 3D structure changes during MD simulation. The homo-oligomers were docked together for observing assembly results by the Cluspro server (Https://cluspro.bu.edu/login.php?redir=/home.php). The PfGCS1-SAPNs were imported to the Cluspro server one by one, in a way that the most optimal state of the assembly at each step that is the SAPN bases were next to each other and antigenic determinants were on top, was assessed in the case of SAPN formation and exposure, respectively.

### Gene cloning, expression, and purification of PfGCS1-SAPN

The designed PfGCS1-SAPN nucleic acid sequence was synthesized and cloned between the *NdeI/XhoI* restriction sites of the pET-24a expression plasmid by Shinegene Company from China. The received lyophilized plasmid was dissolved in Tris-EDTA buffer (10 mM Tris-HCl, pH 8.0, 0.1 mM EDTA) to obtain 40 ng/μl of the recombinant plasmid. Then, 40 ng of plasmid was transformed into the *E*. *coli* DH5α competent cells, and the transformed cells were incubated overnight at 37°C on an LB agar pellet containing kanamycin antibiotic (25 μg/ml). Then, the recombinant plasmid was extracted with the Qiagen plasmid purification kit (Qiagen, Hilden, Germany) and digested with *NdeI/XhoI* restriction enzymes to confirm the presence of insert. Then, the recombinant plasmid was re-sequenced and compared with the designed PfGCS1-SAPN nucleic acid sequence.

For expression of the PfGCS1-SAPN, the recombinant plasmid was transformed into the *E*. *coli* Bl21(DE3), and the transformants were grown in the LB broth culture medium containing kanamycin (25 μg/ml). When the OD_600_ reached 0.6, the bacterial suspension was induced for expression by 0.2mM Isopropyl β-D-1-thiogalactopyranoside (IPTG, Thermo Scientific, USA). After incubation of the induced culture at 37°C for 4 hours, the cell pellet was collected by centrifugation at 6000 rpm for 15 min at 4°C and kept at -20°C until use. The cell pellet was analyzed on 12% SDS-PAGE, and after confirming the presence of PfGCS1-SAPN bands on the gel, purification of the expressed protein was performed with Ni^2+^ nitrilotriacetic acid (Ni-NTA) agarose (Qiagen, Germany) by immobilized metal affinity chromatography (IMAC).

For purifying, after resuspending the cell pellet in denaturation buffer (8 M urea, 20 mM Tris-HCl, 750 mM NaCl, pH 7.9) and incubating at 4°C for 90 min by gentle shaking at 50 rpm, the sonication was performed 20 pulses at 70-s intervals and 75% amplitude in 5 cycles (Ultraschallprozessor, Deutschland, Germany). After centrifuging the bacterial lysate, the supernatant was incubated with Ni-NTA at RT for 2 hours, and then the resin was washed with a 10-column volume of wash buffer (8 M urea, 20 mM Tris-HCl, 750 mM NaCl, and 5 mM imidazole, pH 7.9). Finally, the bound protein was eluted with elution buffer (8 M urea, 20 mM Tris-HCl, 500 mM NaCl, 0.2% SDS, 10 mM 2-mercaptoethanol, 5% glycerol, pH 7.5) at 93°C by heating for 5 min in a bain-marie. The eluates were run on SDS-PAGE to observe the presence of PfGCS1-SAPN bands on the gel, and then western blotting was performed using anti-His antibodies (Qiagen, Germany) to validate the existence of expressed PfGCS1-SAPNs.

### Dialysis to form the protein nanoparticles

For spontaneous assembly, the purified PfGCS1-SAPN solution was poured into 12 kDa dialysis bags and then dialyzed with a decreasing slope of urea concentration to 120 mM and reducing the concentration of SDS and 2ME in dialysis solutions to zero. There are four dialysis solutions, which are shown in Table 1. At first, the dialysis tube was floated at RT for 2 hours into solution I. Dialysis was followed by solutions II and III for further 2 hours in each buffer. Then, the dialysis tube was transferred to solution IV and incubated at 4°C overnight.

**Table 1:** Dialysis solutions for assembling of PfGCS1-SAPNs and NC-SAPNs into SAPNs.

	Urea	Tris	SDS	2ME	NaCl	Glycerol
**Solution I (pH:8)**	6M	20mM	0.05mM	10mM	0.5M	5%
**Solution II (pH:8)**	4M	20mM	-	10mM	0.5M	5%
**Solution III (pH:8)**	2M	20mM	-	5mM	0.5M	5%
**Solution IV (pH:8)**	120mM	20mM	-	-	0.5M	5%

### Analysis of SAPN diameter and shape

Different tests were performed to confirm the correct formation of SAPNs and determine their size in the acceptable range. The diameter size of the SAPNs formed by PfGCS1-SAPNs was measured by using DLS test. The final shape of SAPNs and their diameter size were shown by FESEM.

### Gene cloning, expression, and purification of the negative control SAPN (NC-SAPN)

The NC-SAPN was cloned, expressed, and purified to use as the negative control of the ELISA experiment. For this purpose, the forward and reverse primers were designed and analyzed by Genome Compiler v.2.2.88 to amplify the NC-SAPN gene (accession number: OM937278) containing the basis of SAPN (pentamer and trimmer) and PADRE from the synthesized PfGCS1-SAPN gene. For cloning in pQE30 plasmid, the *BamH*I and *Sma*I restriction sites were considered for forward and reverse primers, respectively. The PCR product was gel purified using a DNA gel extraction kit (Qiagen, Germany) and then ligated to the pGEM T-easy vector and transformed to *E*. *coli* DH5α cells. The presence of the target gene was screened in transformed clones by colony PCR, as well as plasmid extraction followed by enzymatic digestion by *Bam*HI and *Sma*I restriction enzymes. Subsequently, the NC-SAPN sequence was sub-cloned in pQE-30 plasmids and transformed to *E*. *coli* M15 expression host. The obtained clones were grown on LB agar medium with kanamycin (25 μg/ml) and ampicillin (100 μg/ml), and were confirmed to have the desired gene by colony-PCR and digestion with *Bam*HI and *Sma*I restriction enzymes. The recombinant plasmid was sequenced to validate the sequence of the inserted gene.

For expression of the NC-SAPN, *E*. *coli* M15 cells containing pQE-30-NC-SAPN plasmids were induced as mentioned above for PfGCS1-SAPN, and then, the cell lysates were analyzed by SDS-PAGE and Western blotting with anti-His antibodies (Qiagen, Germany) to validate the presence of target protein. The expressed protein was purified as mentioned above for PfGCS1-SAPN, and the eluted proteins were dialyzed like PfGCS1-SAPN dialysis to form the SAPN structure without antigens.

### ELISA

ELISA test was performed to evaluate and confirm the antigenicity of SAPNs formed by PfGCS1-SAPNs and the non-antigenicity of SAPNs formed by NC-SAPNs and also to confirm the presence of the target antigens on the surface of SAPNs formed by PfGCS1-SAPNs. To this end, first, to find the positive plasma samples which have anti-PfGCS1 antibodies, an ELISA was performed using plasma samples (n = 171) from *P*. *falciparum*-infected patients in Chabahar, Sistan and Baluchistan Province in the south-eastern Iran (2005–2010), which is malaria-endemic area. The *P*. *falciparum* infection was confirmed by the molecular diagnosis of the 18ssrRNA gene using the nested-PCR technique, as described previously [36]. Additionally, before blood collection, an informed consent was obtained from adults or parents or legal guardians of children who were participant in this survey. This study was verified and approved by the Ethical Review Committee of Research of the Pasteur Institute of Iran [IR.PII.REC.1399.071]. For this experiment, recombinant PfGCS1 antigen (aa: 176–195 (cd loop) fused to aa: 311–609 of PfGCS1 antigen containing HAP2-GCS domain) was expressed and purified using Ni-NTA agarose, with the method described previously [37]. rPfGCS1 antigen was diluted in a coating buffer (in 0.06 M carbonate bicarbonate buffer, pH 9.6), coated in microplates (100 ng/well), and incubated at 4° C overnight. After washing with 1x PBS-Tween 0.05% (PBS-T), blocking with 2% BSA, and washing again, plasma samples were added at a 1:600 dilution and incubated at RT for 2 hours. After the washing, an anti-human IgG antibody conjugated with horseradish peroxidase (HRP) (Sigma-Aldrich, USA) was added at a 1:25000 dilution and incubated at RT for 1 hour. After the washing step, the bound antibodies were visualized by adding the TMB (Sigma-Aldrich, USA) as the substrate. Next, 2N H_2_SO_4_ was added after ~10 min to stop the reaction, and OD was read at 450 nm using an ELISA microplate reader (BioTek, USA). Fifteen plasma samples from non-exposed and healthy individuals from outside malaria-endemic regions were used as negative controls. The cut-off value was measured as the mean OD of negative samples plus three standard deviations (SD). Those samples that had an OD higher than the cut-off value were considered positive responders with anti-PfGCS1 antibodies.

Afterward, to evaluate the antigenicity of PfGCS1-SAPN, both SAPNs (PfGCS1-SAPN and NC-SAPN) were coated in separate microplates (100 ng/well), simultaneously. After blocking, 30 plasma samples from PfGCS1 seropositive *P*. *falciparum*-infected patients, 30 plasma samples from PfGCS1 seronegative *P*. *falciparum*-infected patients, and 15 plasma samples from healthy individuals from outside malaria-endemic regions (non-exposed individuals) were added as first antibodies (1:800 dilution based on the checkerboard ELISA). After washing, the anti-human IgG antibody conjugated with horseradish peroxidase (1:35000 dilution) (Sigma-Aldrich, St. Louis, MO, USA) was added as the secondary antibody and incubated at RT for 1 hour. Then, after washing the plates with 1xPBS-tween 0.05%, the bound antibodies were visualized by adding the TMB (Sigma-Aldrich, USA) as the substrate. Next, 2N H_2_SO_4_ was added after ~10 min to stop the reaction, and OD was read at 450 nm using an ELISA microplate reader (BioTek, USA). The cut-off value was measured as the mean OD_450nm_ plus three standard deviations (SD) of negative samples (non-exposed individuals, n = 15) to determine the positive samples that have antibodies against PfGCS1-SAPN. The plasma samples with an OD_450nm_ value higher than the cut-off were considered positive responders to PfGCS1-SAPN.

### Statistical analysis

A database was generated with SPSS 23.0 on the Microsoft Windows operating system (SPSS Inc., USA). The Spearman’s Rank Correlation test was used to assess the association between antibody levels to rPfGCS1 and age. The non-parametric Wilcoxon Signed-Rank test was performed to compare the difference in antibody levels to rPfGCS1 and PfGCS1-SAPN. The McNemar test was utilized to evaluate differences between IgG-positive subject proportions for the rPfGCS1 and PfGCS1-SAPN antigens. In addition, to compare the antibody levels to PfGCS1-SAPN in PfGCS1 seropositive and seronegative individuals, the Mann-Whitney test was performed. A p-value less than 0.05 was considered statistically significant.

## Results

### Sequence design of PfGCS1-SAPN and predicting its secondary and tertiary structure

The designed PfGCS1-SAPN amino acid sequence is shown in Fig 1A to depict its different parts. Among all the examined literature oligomers examined by Quark server, choosing the modified Cartilage Oligomeric Matrix Protein (COMP) sequence as pentameric domain [21] and the trimeric domain of Wahome et al. [35] was the best combination for creating the desired 3D structure of PfGCS1-SAPN as is shown in Fig 1C. A GG sequence was chosen as a linker between two oligomeric domains and a GGSG sequence as a linker between PADRE and HAP2-PfGCS1 domains because they offer flexibility to structure. The 3D structure took the desired form, with the base of the SAPN forming the desired shape and the antigens at the two vertices of its basis. As shown in Fig 1B according to the Quark server report on the protein secondary structure, the PfGCS1-SAPN base has the alpha helix as its secondary structure.

### Verifying coiled-coil forming oligomeric domains in PfGCS1-SAPN

**Fig 2:**
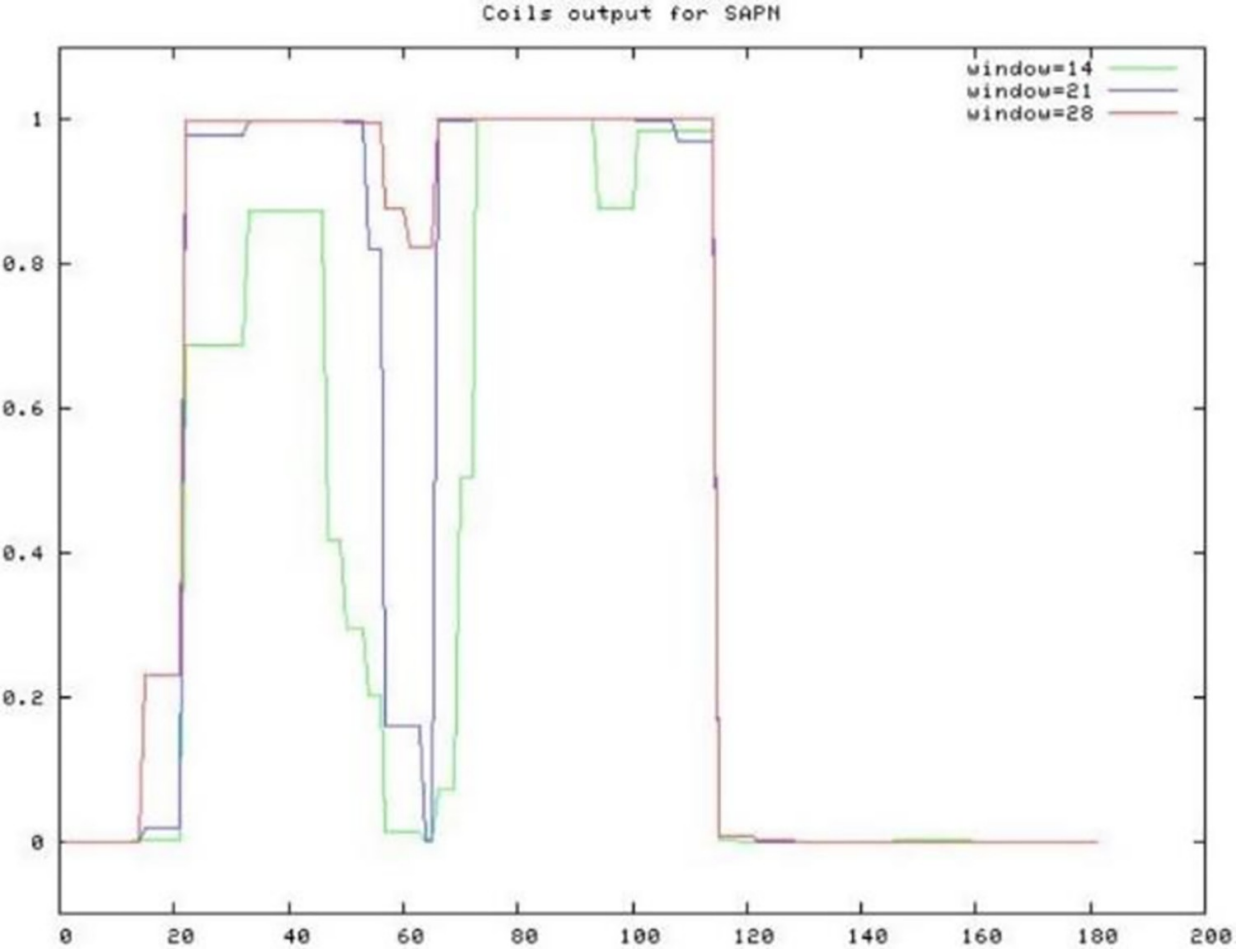
Prediction of coiled-coil pentameric and trimeric regions of PfGCS1-SAPN amino acid sequence. Reading frames of 14, 21, and 28 amino acids are colored in green, blue and red, respectively. In all reading frames, in the pentameric and trimeric regions of the designed PfGCS1-SAPN sequence, the presence of coiled coils is predicted, but in reading frames of 21 and 28 amino acids, the probability of coiled-coil presence is close to one.

**Fig 3:**
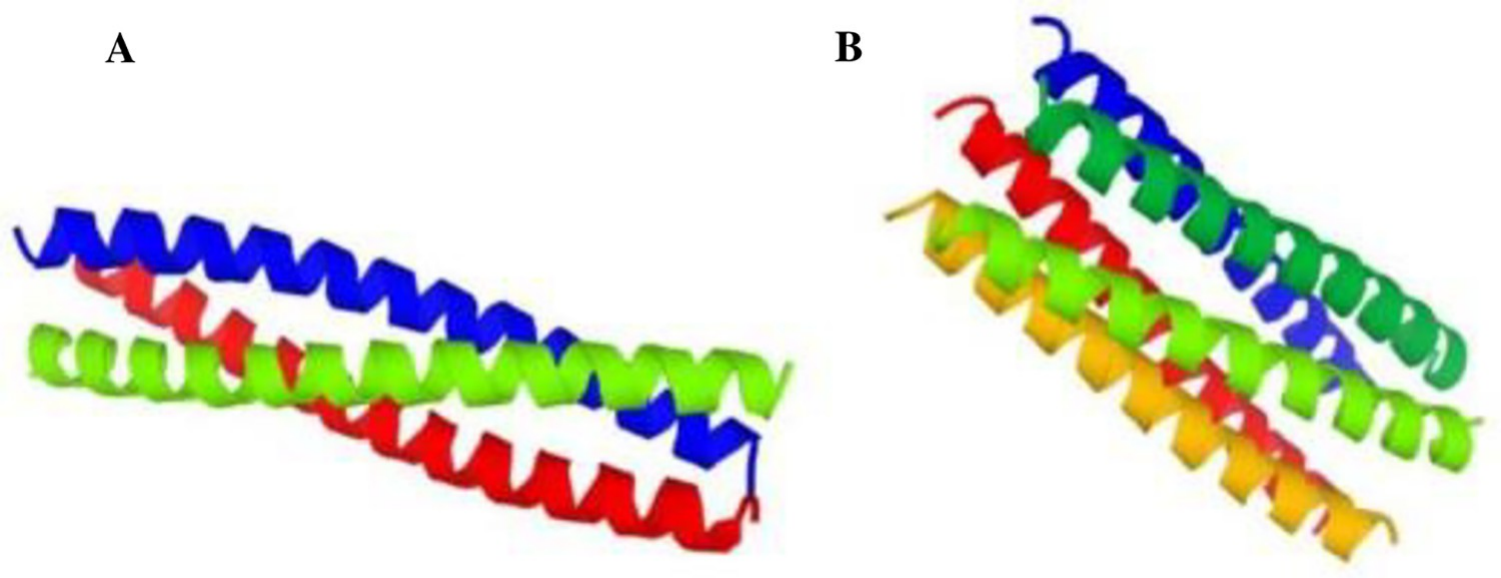
Number of predicted homomers for pentameric (**A**) and trimeric (**B**) parts of PfGCS1-SAPN.

The ExPASy COILS program results showed that two coiled-coil forming fragments are present in the designed PfGCS1-SAPN amino acid sequence (Fig 2). For the pentameric part in reading frames of 21 and 28 amino acids and for the trimeric part in all three reading frames, the probability for coiled-coil fragment forming is close to one (Fig 2). In addition, the GalaxyHomomer server results confirmed that the number of homomers for the designed pentameric and trimeric domains are 5 and 3, respectively (Fig 3).

### Characteristics analysis of PfGCS1-SAPN

The ExPASy ProtParam and Protein-Sol server results for physicochemical characteristics of PfGCS1-SAPN are shown in Table 2. The approximate weight of the PfGCS1-SAPN is about 20kDa. The isoelectric point (pI) is 9.14. The estimated half-life is 30 hours in mammalian reticulocytes (*in vitro*), more than 20 hours in yeast (*in vivo*), and more than 10 hours in *E*. *coli* (*in vivo*). The hydropathicity, aliphatic, and solubility indices were -0.421, 93.35, and 0.562, respectively, and the aliphatic index of 93.35 indicates a positive factor to increase the thermostability of the protein [38]. The solubility index of 0.562 shows that our peptide is soluble in water because the solubility value of 0.45 is considered the solubility threshold, and values above that have a higher solubility than the average soluble *E*. *coli* proteins. Hydropathicity is -0.421, which implies our protein is more hydrophilic than hydrophobic. The grand average of hydropathicity (GRAVY), extinction coefficient considering cystines, and extinction coefficient considering cysteines are -0.421, 19940, and 20190, respectively.

**Table 2:** Physico-chemical characteristics of PfGCS1-SAPN.

Sequence	Number of aa^1^	MW^2^ (Da)	pI^3^	Extinction coe*ffi*cient (with/ without Cys)^4^	Half-life^5^	Alphatic index	GRAVY^6^	solubility index
PfGCS1-SAPN	182	21049.32	9.14	19940/20190	30 h, > 20, > 10	93.35	-0.421	0.562

^1^ aa: amino acid.

^2^ MW: molecular weight.

^3^ pI: isoelectric point.

^4^ Extinction coefficients are in units of M-1 cm-1, at 280 nm measured in water.

^5^ mammalian reticulocytes (*in vitro*), yeast (*in vivo*), *E*. *coli* (*in vivo*).

^6^ GRAVY: Grand average of hydropathicity.

Calculated PfGCS1-SAPN antigenicity and allergenicity characteristics values by VaxiJen and AlgPred servers were 0.5271 and -0.8813, respectively. According to these servers, the threshold values of antigenicity and allergenicity characteristics are 0.4 and -0.4, respectively. Therefore, PfGCS1-SAPN had antigenic characteristics but no allergenic characteristics.

### PfGCS1-SAPN tertiary structure stability

**Fig 4:**
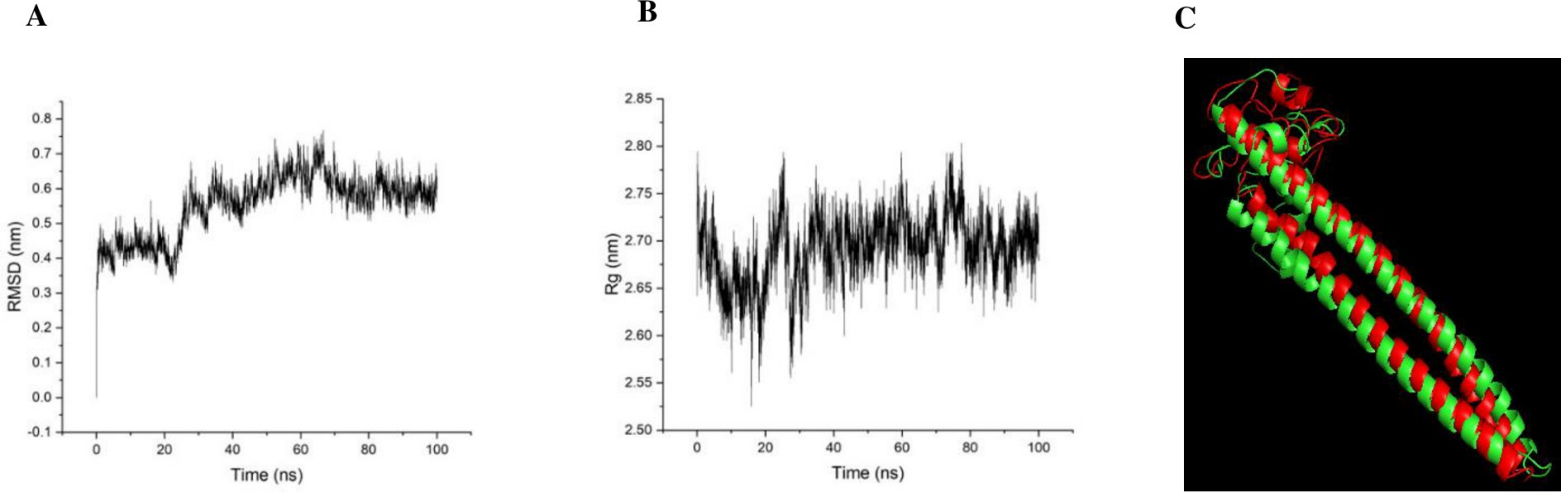
Molecular dynamics simulation of PfGCS1-SAPN by GROMACS 2020. (**A**) All atoms RMSD plot for MD simulation of the PfGCS1-SAPN, (**B**) All atoms Rg plot for MD simulation of PfGCS1-SAPN. RMSD and Rg analyzed plots are for 100 ns MD simulation of PfGCS1-SAPN. (**C**) Superimposition of the predicted (initial 3D structure for MD) and simulated 3D structures (representative 3D structure after clustering) of PfGCS1-SAPN.

First, to verify the stability of the Quark server’s predicted 3D structure, RMSD and Rg analyzes were performed after 100 ns of MD simulation. In the RMSD analysis chart, the fluctuations were ascending for about 75 ns and reached a plateau, then remained constant until the end of 100ns. The average for RMSD in this 100 ns MD run was 0.554 **±** 0.087 (Fig 4A). To further confirm its stability, the Rg was calculated as a measure of compactness (Fig 4B). As can be seen, (Fig 4), the graph has reached a plateau since 40 ns, with no significant difference from the beginning of the simulation. The mean value for the Rg was 2.691 ***±*** 0.035.

The comparison of predicted and simulated PfGCS1-SAPN 3D structures has been shown in Fig 4C by superimposition. The superimposition of predicted and simulated 3D structures shows that the initial PfGCS1-SAPN 3D structure has not undergone significant changes after MD simulation and has retained its original form.

**Fig 5:**
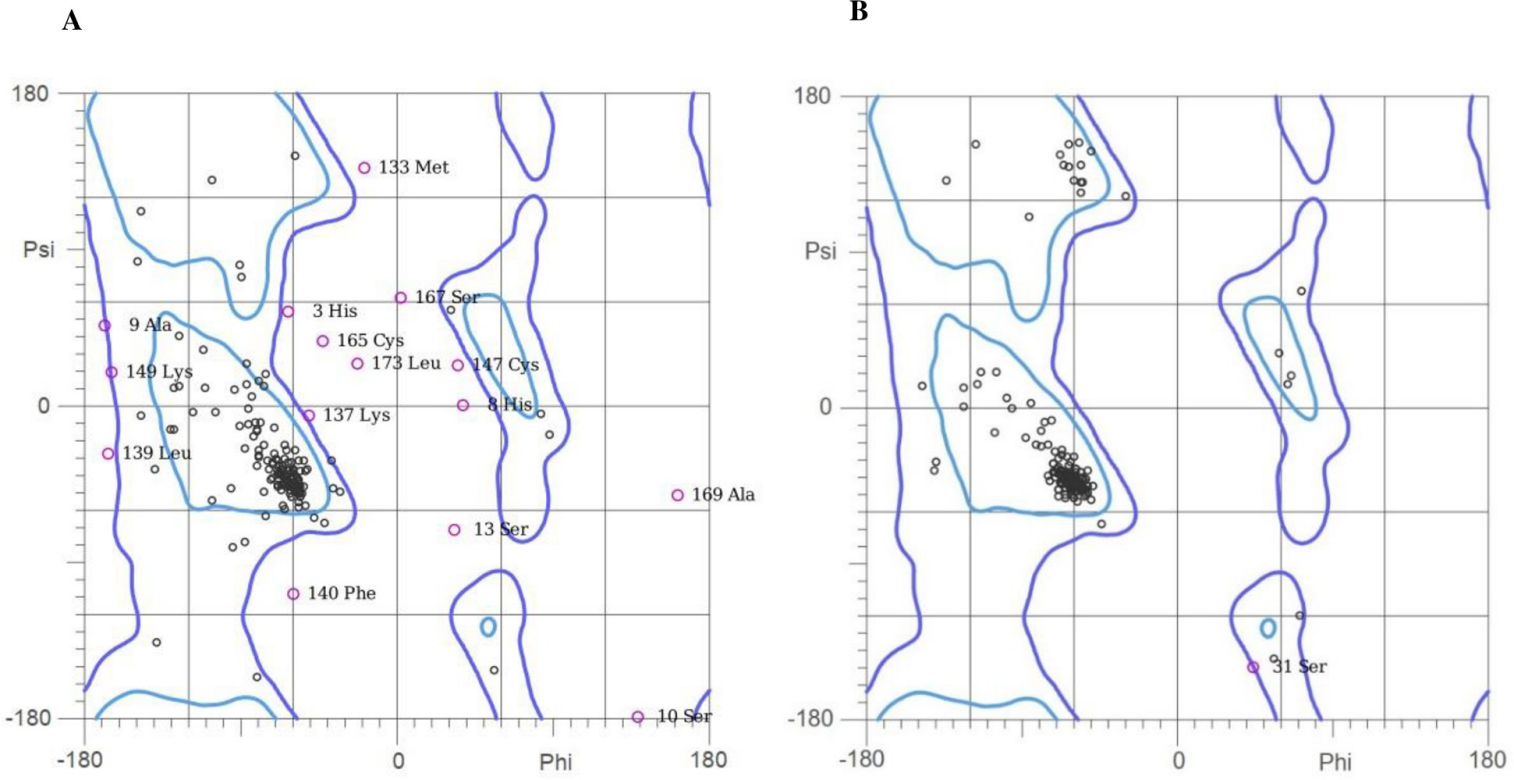
Validation of predicted (**A**) and simulated (**B**) PfGCS1-SAPN 3D structure Ramachandran plot using MolProbity server.

Ramachandran plots also were used to validate the stability and accuracy of the protein 3D structure. Ramachandran plot for predicted 3D structure shows that 74.4% (134/180) of all residues were in favored (98%) regions, 87.2% (157/180) of all residues were in allowed (>99.8%) regions, and there were 23 outliers (Fig 5A). While Ramachandran plot for MD simulated 3D structure shows that 95.0% (171/180) of all residues were in favored (98%) regions, 99.4% (179/180) of all residues were in allowed (>99.8%) regions, and there was one outlier (Fig 5B).

### Assembling the PfGCS1-SAPNs

**Fig 6:**
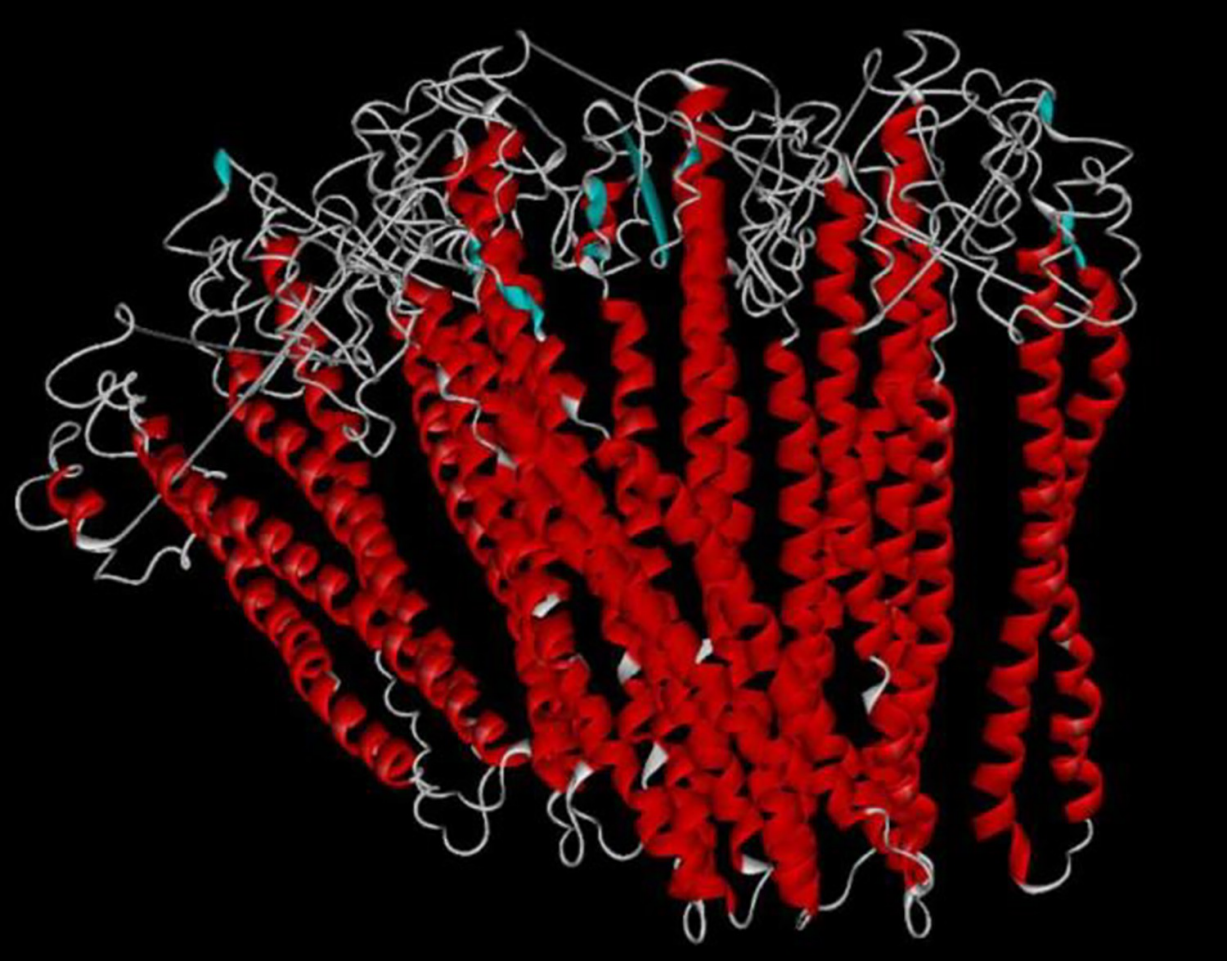
The assembly result of homo-oligomers of PfGCS1-SAPN by the Cluspro server. This representative picture was obtained from the assembly of 11 PfGCS1-SAPNs using docking. According to the results, the SAPN bases were next to each other, and antigenic determinants were on the SAPN surface.

The Cluspro server result for the PfGCS1-SAPNs docking showed that the protein bases were next to each other to form the core of SAPN, and the antigens were on the surface of the assembled structure for the purpose of antigens exposure (Fig 6).

### Expression and purification of the PfGCS1-SAPNs / NC-SAPNs

**Fig 7:**
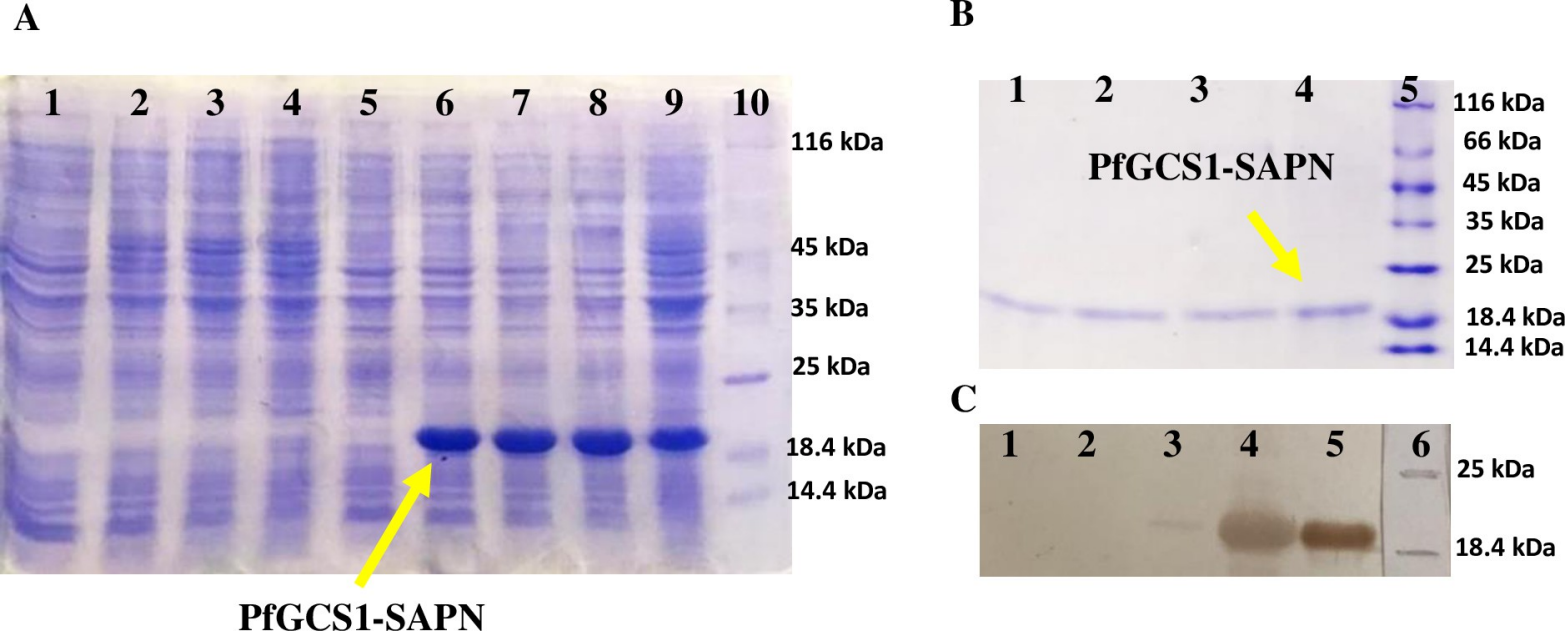
SDS-PAGE and Western blot analysis of PfGCS1-SAPN. (**A**) SDS-PAGE analysis of PfGCS1-SAPN expression. Lanes 1–4: *E*. *coli* BL21(DE3)-pET24a, lanes 5–9: *E*. *coli* BL21(DE3)-PfGCS1-SAPN-pET24a. Lanes 1, 5: Before induction; lanes 2 and 6: 1h after induction with IPTG; lanes 3 and 7: 2h after induction; lanes 4 and 8: 4h after induction; lane 9: 16h after induction; and lane 10: molecular weight protein marker (Fermentas, 116–14.4 kDa). (**B**) SDS-PAGE analysis of purified PfGCS1-SAPN. Lanes 1–4: purified PfGCS1-SAPN. Lane 5: molecular weight protein marker (Fermentas, 116–14.4 kDa). (**C**) Western blot analysis of PfGCS1-SAPN protein with anti-His tag mAb. Lanes 1 and 2: Before induction and 4h after induction of *E*. *coli* BL21(DE3)-pET24a as negative controls, respectively. Lanes 3 and 4: Before induction and 4h after induction of *E*. *coli* BL21(DE3)- PfGCS1-SAPN-pET24a, respectively. Lane 5: purified PfGCS1-SAPN. Lane 6: molecular weight protein marker (Fermentas, 116–14.4 kDa).

The PfGCS1-SAPN gene was successfully cloned in the pET-24a plasmid and expressed in *E*. *coli* Bl21(DE3) host cells. *E*. *coli* Bl21(DE3) cells containing recombinant pET-24a expressed PfGCS1-SAPNs in LB broth with adding an optimal IPTG concentration of 0.2 mM in 0.6–0.8 absorbance of the cells at OD_600nm_. Analysis of the induced cells on SDS-PAGE showed expression of a band with a molecular weight of ~20 kDa at different times (1, 2, 4, and 16 h) after induction (Fig 7A). Furthermore, SDS-PAGE analysis of purified PfGCS1-SAPNs showed a single band (20 kDa) without extra bands of host proteins (Fig 7B). Western blotting analysis using anti-His antibodies confirmed the presence of PfGCS1-SAPNs in both induced cells and purified eluates (Fig 7C).

**Fig 8:**
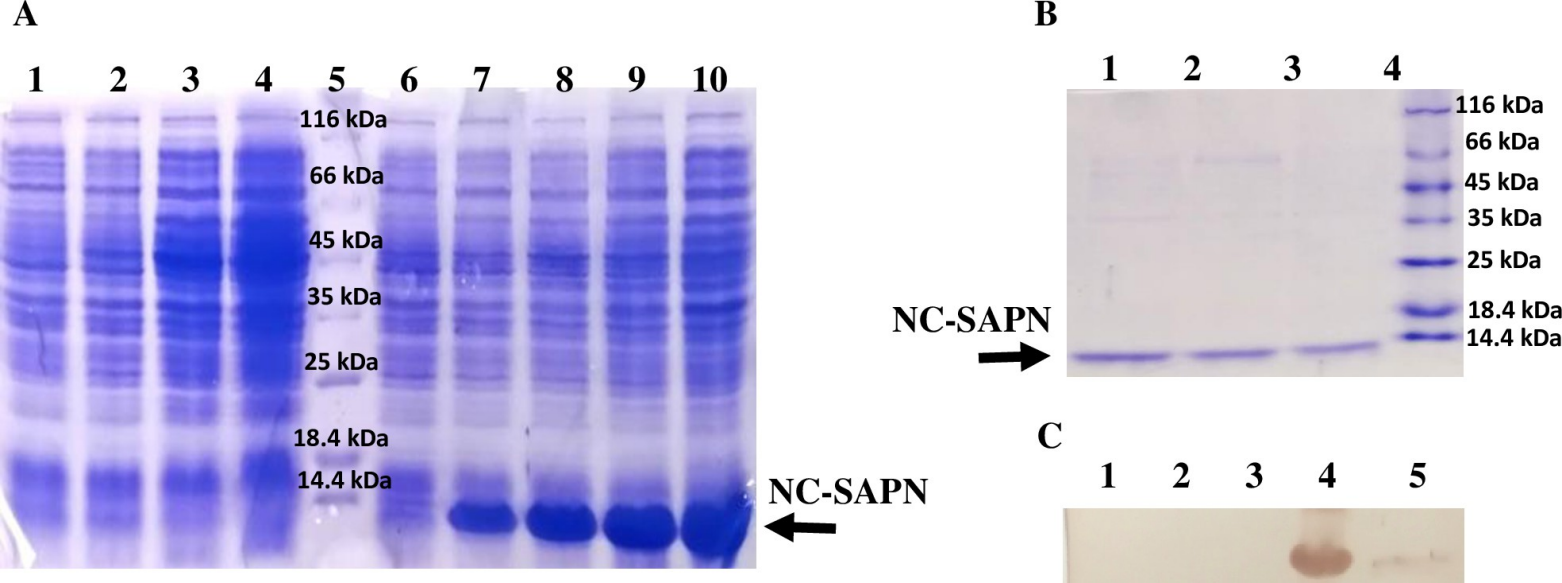
SDS-PAGE and Western blot analysis of NC-SAPN (the basis of nanoparticle). (**A**) SDS-PAGE analysis of NC-SAPN expression. Lanes 1–4: *E*. *coli* M15-pQE30, lanes 6–10: *E*. *coli* M15-NC-SAPN-pQE30. Lanes 1, 6: Before induction; lanes 2 and 7: 1h after induction with IPTG; lanes 3 and 8: 2h after induction; lanes 4 and 9: 4h after induction; lane 10: 16h after induction; and lane 5: molecular weight protein marker (Fermentas, 116–14.4 kDa). (**B**) SDS-PAGE analysis of purified NC-SAPN. Lanes 1–3: purified NC-SAPN. Lane 4: molecular weight protein marker (Fermentas, 116–14.4 kDa). (**C**) Western blot analysis of PfGCS1-SAPN protein with anti-His tag mAb. Lanes 1 and 2: Before induction and 4h after induction of *E*. *coli* M15-pQE30 as negative controls, respectively. Lanes 3 and 4: Before induction and 4h after induction of *E*. *coli* M15-NC-SAPN-pQE30, respectively. Lane 5: purified PfGCS1-SAPN.

The NC-SAPN sequence was successfully cloned and expressed in the *E*. *coli* M15-pQE30 expression system to construct the basis of our SAPN structure without PfGCS1 antigenic epitopes to be used as the negative control in the ELISA test. SDS-PAGE analysis of the induced cells revealed the expression of a band with a molecular weight of ~10 kDa (Fig 8A). After successfully purification, purified NC-SAPNs analysis on the SDS-PAGE showed a single band with the molecular weight of ~10 kDa (Fig 8B). The induced and purified antigens were also confirmed using anti-His antibodies in western blotting analysis (Fig 8C).

### SAPNs formation, diameter and shape determination

**Fig 9:**
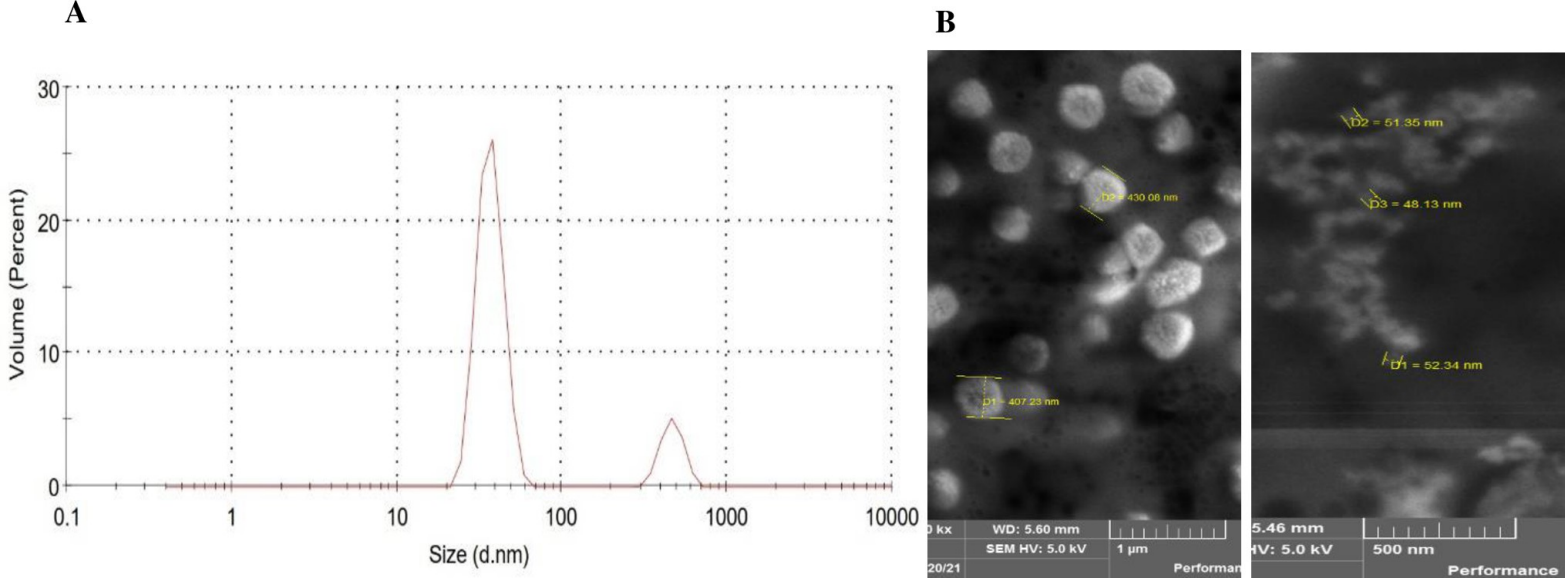
Confirmation of the size and shape of assembled SAPNs by PfGCS1-SAPN using DLS (**A**) and FESEM (**B**) tests, respectively. The diameter of the 85.4% of SAPNs nanoparticles is ideally in the range of 20 to 100 nm, with a peak of ~37.17 nm.

In order to confirm SAPNs formation after dialysis of the PfGCS1-SAPNs, the DLS test reported two peaks of 37.17 nm (25–60 nm) with a volume percentage of 85.4 and 464.5 nm with a volume percentage of 14.6 as the SAPNs diameter in the volume bar chart (Fig 9A).

The FESEM test determined the shape of the SAPNs to be spherical. In this test, due to the presence of two peaks for the SAPNs diameter, two sizes of particles (~37 nm and ~464 nm) were observed by the FESEM device. As a result of low magnification of the FESEM device, the picture of small particles was not with good resolution. Nevertheless, FESEM confirmed the presence of spherical PfGCS1-SANP particles with a mean diameter of 48–52 nm (Fig 9B) which can be generalized by measured particles diameter in DLS with the mean diameter of 37 nm.

### Natural acquired antibody responses to rPfGCS1 antigen in a malaria endemic area of Iran

**Fig 10:**
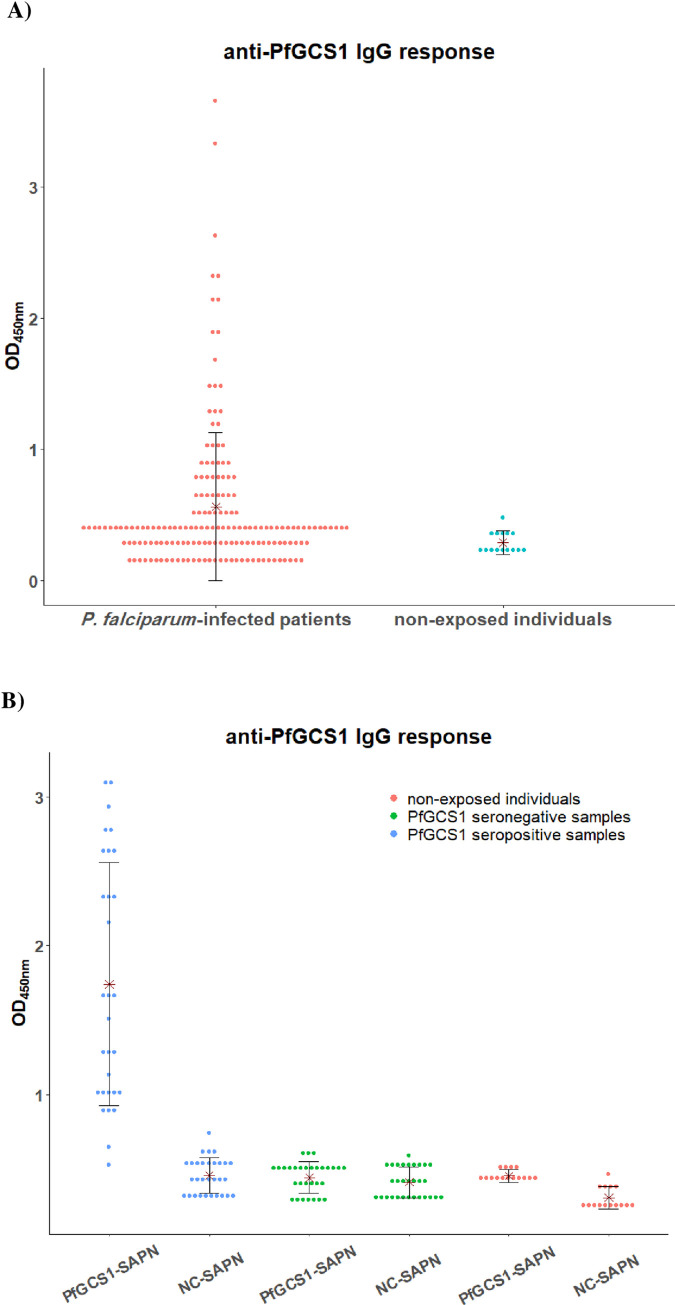
Antibody responses of the *P*. *falciparum-*infected patients to PfGCS1 antigen. A) anti-PfGCS1 IgG antibody responses to rPfGCS1 antigen among plasma samples obtained from Iranian *P*. *falciparum*-infected patients (n = 171) were measured using ELISA. Fifteen plasma samples from non-exposed individuals were used as negative controls. The dots represent the OD_450nm_ for each plasma sample, and the stars and vertical lines indicate the mean ODs and standard deviations (SD) for each group, respectively. The cut-off for the anti-rPfGCS1 IgG antibody was 0.51 (mean of negative controls + 3SD). B) IgG antibody responses against PfGCS1-SAPN and NC-SAPN among PfGCS1 seropositive plasma samples (n = 30), PfGCS1 seronegative plasma samples (n = 30), and non-exposed individuals (n = 15). The dots represent the OD_450nm_ for each plasma sample, and the stars and vertical lines indicate the mean ODs and standard deviations (SD) for each group, respectively. The cut-off for the anti-PfGCS1-SAPN IgG antibody was 0.585 (mean of negative controls + 3SD).

To determine the naturally acquired antibody responses to the rPfGCS1 antigen, the presence of anti-PfGCS1 was evaluated in plasma samples from 171 *P*. *falciparum*-infected patients (aged 4–65 years; mean ± SD = 29.8 ± 13.09 years) from Sistan and Baluchistan Province in Iran. The results revealed that 25.1% (43/171) of samples had positive anti-PfGCS1 IgG antibody responses (Fig 10A). Analysis of the antibody responses to rPfGCS1 showed high (4.1%, OD ≥2), medium (8.8%, 1≤ OD< 2), and low (12.2%, cut-off<OD<1) responses. The levels of anti-PfGCS1 IgG antibodies were not correlated with age (r = 0.062, P = 0.538; Spearman’s correlation test). None of the plasma samples from healthy (non-exposed) individuals had IgG antibodies to the target antigen.

### Confirming the presence of antigenic determinants on the surface of SAPNs

An ELISA test was designed and performed to know whether the target antigenic regions are exposed on the SAPNs surface. For this purpose, 30 out of 43 PfGCS1 seropositive (5 high responder sera, 12 medium responder sera, and 13 low responder sera) and 30 PfGCS1 seronegative samples from *P*. *falciparum-*infected patients were used in ELISA. The results of the ELISA test showed that the PfGCS1 seropositive samples (28/30) were able to recognize SAPNs containing cd loop and HAP2-PfGCS1 domains (PfGCS1-SAPN) with a mean OD_450nm_ ± SD = 1.740 ± 0.817; however, none of the PfGCS1 seronegative samples could recognize PfGCS1-SAPN (mean OD_450nm_ ± SD = 0.440 ± 0.107; P < 0.0001, Mann-Whitney test). Besides, the sera from healthy individuals outside the endemic areas (non-exposed individuals) were not able to recognize SAPNs with or without antigenic determinants (cut-off = 0.585). In addition, none of the PfGCS1 seropositive or seronegative samples were able to recognize SAPNs without antigens (mean OD_450nm_ ± SD = 0.455 ± 0.121 and 0.409 ± 0.104, respectively). These findings indicate that, first, antigenic epitopes are located on the SAPN surface and, second, positive sera specifically recognize antigenic epitopes of the PfGCS1-SAPN and are unable to identify the NC-SAPN (Fig 10B).

### Comparative analysis of the naturally acquired antibody responses to rPfGCS1 and PfGCS1-SAPN antigens

**Fig 11:**
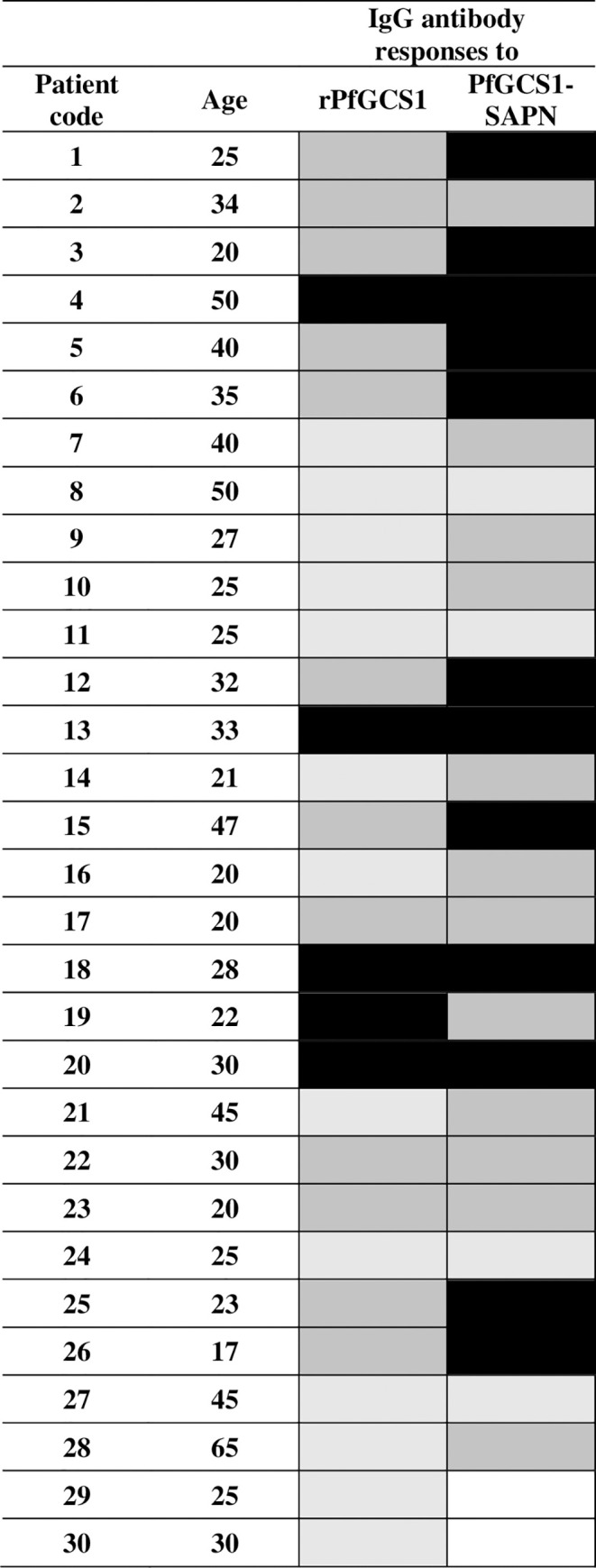
The comparison pattern of total IgG responses to rPfGCS1 and PfGCS1-SAPN antigens among 30 individuals. Ages are given in years. The cut-off values were 0.51 and 0.585 for rPfGCS1 and PfGCS1-SAPN antigens, respectively. The OD mean values have been divided into the following groups: OD ≥ 2: High-positive antibody responses (black), 1 ≤ OD < 2: Medium-positive responses (dark gray), cut-off < OD < 1: Low-positive responses (pale gray), and OD < cut-off: Negative (white).

Among 30 PfGCS1 seropositive individuals, 28 sera had IgG antibodies to PfGCS1-SAPN. This difference in the proportion of responders to rPfGCS1 and PfGCS1-SAPN was not statistically significant (P = 0.5, McNemar test, Fig 11). The level of anti-PfGCS1-SAPN IgG antibodies (mean OD_450nm_ ± SD = 1.740 ± 0.817) was significantly higher than anti-rPfGCS1 (mean OD_450nm_ ± SD = 1.248 ± 0.557) IgG antibodies (P = 0.001, Wilcoxon test, Fig 11). Interestingly, the examined sera had heterogeneity in their responses to PfGCS1-SAPN and rPfGCS1 antigens. Half of the sera (n = 15) had higher levels of anti-PfGCS1-SAPN IgG antibodies compared with anti-rPfGCS1 IgG antibodies; however, one serum had lower levels, and two sera had no anti-PfGCS1-SAPN IgG antibodies (Fig 11).

## Discussion

There is no effective vaccine for malaria as one of the most severe infectious diseases. Even the leading malaria vaccine, RTS,S (a protein-based vaccine), provides a low percentage of effectiveness and does not fulfill a long-lasting immunity [2, 39]. Since the protein vaccines are weak immunogens, effective adjuvants are necessary to increase their immunogenicity. Therefore, one of the limitations of protein-based vaccines is the selection of co-administered adjuvants [40]. SAPN, as a promising platform, is capable of designing effective vaccines to expose antigenic epitopes against diverse pathogens without the need for adjuvants [22, 41, 42]. There are various SAPN-based vaccines in literature, which confirm its efficacy against several pathogens, including *P*. *berghei* [22], *P*. *falciparum* [43–45], severe acute respiratory syndrome (SARS) coronavirus [21], *Toxoplasma gondii* [46], and human immunodeficiency viruses (HIV) [35]. In the current investigation, a SAPN vaccine containing antigenic determinants of PfGCS1 as a potential malaria vaccine was designed, produced, and confirmed *in silico* and *in vitro* in terms of size, shape, and the presentation of PfGCS1 epitopes on its surface.

In this study, for the first time, the antigenic determinants of PfGCS1 antigen called cd loop and HAP2-PfGCS1 are used in designing our SAPN substructure called PfGCS1-SAPN to aim for transmission-blocking immunity against malaria. These two sequences are highly conserved that promote zygote formation and malaria transmission [25–29]. Although PfGCS1 is an essential antigen in fertilization, previous studies showed that the antibodies induced against it were not strong enough in inhibiting oocyst formation [30, 31]. Therefore, it seems that using these antigens in the SAPN scaffold could better deliver epitopes to the immune system and lead to better immune responses. Since the use of the universal T-helper epitopes in vaccine design is a great help in increasing immunogenicity, and due to the effectiveness of the PADRE T-helper epitope in eliciting the immune response [21, 22, 44–47], this sequence was also used in PfGCS1-SAPN design to enhance the immunogenicity, that remains to be tested in future studies. Similar to previous studies [21, 22, 35, 43–46, 48, 49], the designed amino acid sequence was confirmed to form the SAPN by assembling the monomers and surface exposure of antigenic determinants as confirmed by 3D structure prediction and assembly analysis. Besides, antigenic determinants are expressed on the surface of SAPNs formed by PfGCS1-SAPNs as verified by the ELISA.

The prediction of physicochemical properties of the designed PfGCS1-SAPN showed that pI implies the alkaline feature and the positive electrical charge of the PfGCS1-SAPN at the pH of the blood and also solutions used throughout the laboratory process. One of a protein’s key characteristics is its half-life, i.e., the time at which only half of the newly generated protein is still present in cells. The protein half-life shows the stability of the target protein, and the estimated half-life indicates the stability of the PfGCS1-SAPN in mammals, which means enough time to start the immune system activation. Other properties showed that PfGCS1-SAPN is a soluble protein, which tends to be a little more hydrophilic than hydrophobic. The high amount of the extinction coefficient predicted by the ExPASy ProtParam tool was justified and confirmed due to the high efficiency of PfGCS1-SAPNs expression and their thick bands on an SDS-PAGE gel. A comparison of threshold limits and calculated values of antigenicity and allergenicity confirmed that the PfGCS1-SAPN has antigenicity but no allergenicity. The PfGCS1-SAPN sequence gained its desired 3D structure by its pentameric and trimeric oligomerization domains, and the secondary structure of the pentameric and trimeric oligomerization domains is alpha-helix which is required to form coiled-coil structures. This 3D structure and the placement of oligomeric parts and antigenic parts became similar to the designed 3D structure in similar studies [21, 22, 35, 43–46, 48–51].

Reaching the calculated RMSD plot by GROMACS 2020 at a plateau and remaining constant indicated that the 3D structure is in its stable state and has maintained its stability in a long MD simulation as in Doll et al. [51]. The stability of the PfGCS1-SAPN 3D model was confirmed strongly by considering the low mean value of the RMSD in achieving the steady-state according to the fact that the initial simulation structure was obtained non-experimentally by the QUARK server. The superimposition of the predicted and simulated 3D structures also demonstrated that predicted 3D structure has high accuracy and stability, as can be seen in Doll et al. [51]. Ramachandran results for predicted and simulated 3D structures Showed that the number of residues in favored regions and allowed regions increased from 74.4% to 95.0% and from 87.2% to 99.4%, respectively, and also the number of outlier residues decreased from 23 to 1, indicating a correction of the predicted 3D structure during the MD simulation.

After cloning and expressing the PfGCS1-SAPN gene in pET-24a, the PfGCS1-SAPN bands were thick, which indicates the high efficiency of PfGCS1-SAPN production in the *E*. *coli* BL21 (DE3) host expression cells. During purification, imidazole did not isolate PfGCS1-SAPN from Ni-NTA resins, which could be due to other bonds between PfGCS1-SAPNs and resins, such as hydrophobic bonds, other than the binding of the His-tag and Ni-NTA. Adding 2ME and SDS in elution buffer and heating at 93°C for 5 min solved this problem and separated the protein from Ni-NTA. Confirmation of the PfGCS1-SAPN bands’ presence for expressed and purified samples by Western blotting conclusively indicated that the PfGCS1-SAPNs were expressed and purified. Considering that after DLS analysis on the dialyzed SAPNs, 85% of the SAPNs have a diameter of 37.17 nm, it can be said with great confidence that the SAPN particles were in the ideal range of 20 nm to 100 nm, as had been mentioned in the literature [41, 52, 53]. Furthermore, the sphericity of SAPN formed by PfGCS1-SAPN was also confirmed by FESEM similar to other studies [21, 22, 35, 43–46, 48–51]. The presence and accessibility of epitopes on the SAPN surface and the antigenicity evaluation of epitopes were confirmed by ELISA analysis, as described previously [21, 22, 35, 43–45]. In addition, none of the sera (containing anti-PfGCS1 antibodies) could recognize the NC-SAPN by ELISA. Furthermore, the reactivity of sera between rPfGCS1 and PfGCS1-SAPN was compared, and the results showed that most sera had higher reactivity to PfGCS1-SAPN, implying the appropriate conformation of the epitopes in PfGCS1-SAPN. Only two samples with a low response to rPfGCS1 (near borderline) did not have antibodies against PfGCS1-SAPN. This result may be due to the antigenic determinants used in PfGCS1-SAPN. It is possible that the two mentioned samples had antibodies against epitopes other than those used in PfGCS1-SAPN. Altogether, these results suggest the sensitive and specific binding of PfGCS1-specific antibodies to the SAPNs formed by PfGCS1-SAPNs.

## Conclusion

In conclusion, in order to design the PfGCS1-SAPN sequence to form SAPN, two determinants of PfGCS1, called cd loop and HAP2-PfGCS1, and PADRE T-helper epitope were integrated to a basis containing two coiled-coil oligomerization domains. *In silico* and *in vitro* methods were applied to confirm the assembly of SAPN from the PfGCS1-SAPNs. It has been shown that SAPN formed by PfGCS1-SAPN has been recognized by antibodies in malaria patient sera, indicating developing an efficient new nanovaccine against *P*. *falciparum*. Although the *in vivo* tests have remained to be examined in the future, the analyses conducted for PfGCS1-SAPN showed that this nanovaccine is produced in the correct form and is recognized by naturally acquired antibodies in *P*. *falciparum*-infected patients, confirming it as a potential malaria vaccine candidate.

## Supporting information

S1 Raw image (PDF)
