# Supplementary material for: Design and development of a self-assembling protein nanoparticle displaying PfHAP2 antigenic determinants recognized by natural acquired antibodies
Source: PLoS One. 2022 Sep 12;17(9):e0274275. doi: 10.1371/journal.pone.0274275 (PMC9467374; doi:10.1371/journal.pone.0274275)
Supplement: S1 Raw image — (PDF) [file pone.0274275.s001.pdf]

Figure 7.

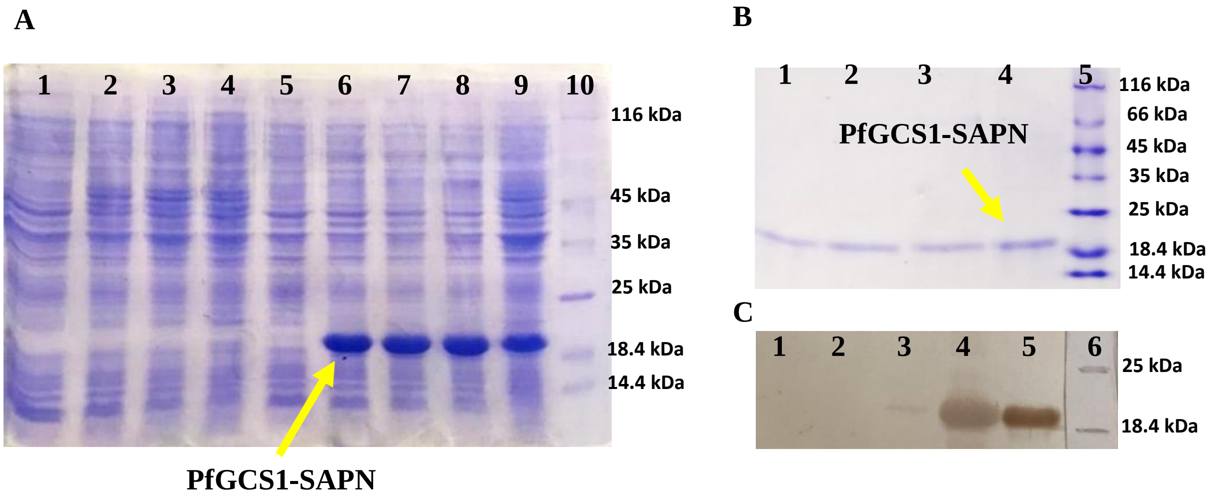

Fig. 7 SDS-PAGE and Western blot analysis of PfGCS1-SAPN. (**A**) SDS-PAGE analysis of PfGCS1-SAPN expression. Lanes 1-4: *E. coli* BL21(DE3)-pET24a, lanes 5-9: *E. coli* BL21(DE3)-PfGCS1-SAPN-pET24a. Lanes 1, 5: Before induction; lanes 2 and 6: 1h after induction with IPTG; lanes 3 and 7: 2h after induction; lanes 4 and 8: 4h after induction; lane 9: 16h after induction; and lane 10: molecular weight protein marker (Fermentase, 116-14.4 kDa). (**B**) SDS-PAGE analysis of purified PfGCS1-SAPN. Lanes 1-4: purified PfGCS1-SAPN. Lane 5: molecular weight protein marker (Fermentase, 116-14.4 kDa). (**C**) Western blot analysis of PfGCS1-SAPN protein with anti-His tag mAb. Lanes 1 and 2: Before induction and 4h after induction of *E. coli* BL21(DE3)-pET24a as negative controls, respectively. Lanes 3 and 4: Before induction and 4h after induction of *E. coli* BL21(DE3)- PfGCS1-SAPN-pET24a, respectively. Lane 5: purified PfGCS1-SAPN. Lane 6: molecular weight protein marker (Fermentase, 116-14.4 kDa).

Figure 8.

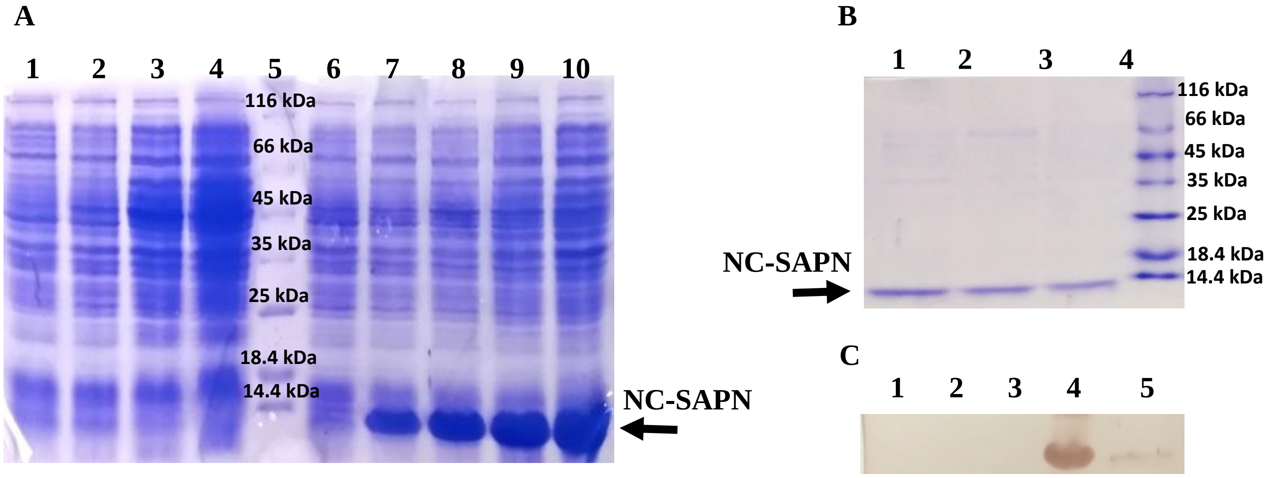

Fig. 8 SDS-PAGE and Western blot analysis of NC-SAPN (the basis of nanoparticle). (**A**) SDS-PAGE analysis of NC-SAPN expression. Lanes 1-4: *E. coli* M15-pQE30, lanes 6-10: *E. coli* M15-NC-SAPN-pQE30. Lanes 1, 6: Before induction; lanes 2 and 7: 1h after induction with IPTG; lanes 3 and 8: 2h after induction; lanes 4 and 9: 4h after induction; lane 10: 16h after induction; and lane 5: molecular weight protein marker (Fermentase, 116-14.4 kDa). (**B**) SDS-PAGE analysis of purified NC-SAPN. Lanes 1-3: purified NC-SAPN. Lane 4: molecular weight protein marker (Fermentase, 116-14.4 kDa). (**C**) Western blot analysis of PfGCS1-SAPN protein with anti-His tag mAb. Lanes 1 and 2: Before induction and 4h after induction of *E. coli* M15-pQE30 as negative controls, respectively. Lanes 3 and 4: Before induction and 4h after induction of *E. coli* M15-NC-SAPN-pQE30, respectively. Lane 5: purified PfGCS1-SAPN
